# Determination of a steady-state isotope dilution protocol for carbon oxidation studies in the domestic cat

**DOI:** 10.1017/jns.2023.44

**Published:** 2023-05-29

**Authors:** Julia Guazzelli Pezzali, Jocelyn G. Lambie, Stuart M. Phillips, Anna K. Shoveller

**Affiliations:** 1 Department of Animal Biosciences, Ontario Agricultural College, University of Guelph, Guelph, ON, Canada; 2 Department of Animal Science, Iowa State University, Ames, IA, United States; 3 Department of Kinesiology, McMaster University, Hamilton, ON, Canada

**Keywords:** Amino acid metabolism, Amino acid oxidation technique, Bicarbonate metabolism, Carnivore

## Abstract

The present study aimed to develop an isotope protocol to achieve equilibrium of ^13^CO_2_ in breath of cats during carbon oxidation studies using L-[1-^13^C]-Phenylalanine (L-[1-^13^C]-Phe), provided orally in repeated meals. One adult male cat was used in two experiments. In each experiment, three isotope protocols were tested in triplicate using the same cat. During carbon oxidation study days, the cat was offered thirteen small meals to achieve and maintain a physiological fed state. In experiment 1, the isotope protocols tested (A, B and C) had a similar priming dose of NaH^13^CO_3_ (0⋅176 mg/kg; offered in meal 6), but different priming [4⋅8 mg/kg (A) or 9⋅4 mg/kg (B and C); provided in meal 6] and constant [1⋅04 mg/kg (A and B) or 2⋅4 mg/kg (C); offered in meals 6–13] doses of L-[1-^13^C]-Phe. In experiment 2, the isotope protocols tested (D, E and F) had similar priming (4⋅8 mg/kg; provided in meal 5) and constant (1⋅04 mg/kg; provided in meals 5–13) doses of L-[1-^13^C]-Phe, but increasing priming doses of NaH^13^CO_3_ (D: 0⋅264, E: 0⋅352, F: 0⋅44 mg/kg; provided in meal 4). Breath samples were collected using respiration chambers (25-min intervals) and CO_2_ trapping to determine ^13^CO_2_:^12^CO_2_. Isotopic steady state was defined as the enrichment of ^13^CO_2_, above background samples, remaining constant in at least the last three samples. Treatment F resulted in the earliest achievement of ^13^CO_2_ steady state in the cat's breath. This feeding and isotope protocol can be used in future studies aiming to study amino acid metabolism in cats.

## Introduction

Metabolite concentrations have been used to evaluate the health status of animals and humans, and as a tool to provide an understanding of the complex interplay of metabolism. However, concentrations of metabolites are static measurements and do not reveal important kinetic movement into (appearance) and out of (disappearance) a particular metabolic pool. Unsurprisingly, plasma amino acid (AA) concentrations are poorly correlated with estimates of AA and protein requirements^(1,2)^, and thus, are considered an insensitive method to estimate AA requirements^(3)^. The use of stable isotopes in human and animal research has provided a highly sensitive method to measure kinetics of metabolites^(4)^. Furthermore, the use of stable isotope tracers together with indirect calorimetry (to quantify the volume of CO_2_ produced; VCO_2_) have made it possible to quantify the rate of oxidation of substrates, such as, but not limited to, AA. The measurement of AA oxidation can be used to determine the requirement of AA, where the oxidation of an indicator AA, such as L-[1-^13^C]-Phenylalanine (L-[1-^13^C]-Phe), at varying intakes of the test AA is used as the biological outcome^(5)^. After the invention of IAAO in 1983^(6)^, which was first applied in pigs, the IAAO was subsequently applied in humans using intravenous ^13^C-Phe to determine the Lys requirement^(7)^. The IAAO protocol with oral provision of isotope was then validated^(8)^, making it less invasive than the intravenous approach. This less invasive approach was further supported in a following study^(9)^ in which identical lysine requirement estimates were found in humans repeatedly fed ^13^C-Phe or intravenously supplied ^13^C-Phe. Since then, the IAAO technique has been broadly used under different states of health^(10–12)^ and in different species^(13–17)^ due to its non-invasive and highly sensitive nature.

More recently, we have worked on applying the IAAO technique in adult cats to improve our limited understanding of AA requirements in obligate carnivores and more specifically, the domestic cat. First, we developed a semi-synthetic diet to use in carbon oxidation studies^(18,19)^ and confirmed that enrichment of ^13^CO_2_ can be captured using respiration chambers during an isotope dilution study in cats that received ^13^C-Phe orally rather than intravenously^(20)^. However, cats failed to achieve a steady state of ^13^CO_2_ enrichment in breath using oral priming (4⋅8 mg/kg) and constant (1⋅04 mg/kg) doses of L-[1-^13^C]-Phe^(20)^, which were provided over a thirteen small meal regimen as reported in dogs^(15)^. The oxidation of L-[1-^13^C]-Phe can only be calculated when an equilibrium of ^13^CO_2_ enrichment in breath is reached, which is achieved using the constant infusion-isotope dilution approach. Equilibrium, also referred to as a steady state, is achieved when the rate of appearance of a metabolite in a specific body pool is equal to its rate of disappearance. However, isotopic equilibrium may take several hours to be reached if the pool size of the metabolite is large in relation to its turnover rate^(21)^, which may present practical and ethical concerns. To overcome this challenge, a priming dose of L-[1-^13^C]-Phe is given in conjunction with a constant infusion of L-[1-^13^C]-Phe in carbon oxidation studies in humans^(22)^, pigs^(23)^ and dogs^(15, 24)^. However, this approach only reduces the time to reach the isotopic steady state if the prime-to-constant ratio of the tracer is adequate to the pool size and turnover of the substrate^(25)^. Furthermore, ^13^CO_2_ produced from oxidation of L-[1-^13^C]-Phe enters the bicarbonate pool before exhalation. However, the rate of exchange between ^13^CO_2_ and the unlabelled bicarbonate pool is slow and may delay the time to reach ^13^CO_2_ steady state in breath. Thus, priming the bicarbonate pool may be used as an option to reduce the time to reach the steady state of labelled expired CO_2_^(26)^. The ideal priming dose of bicarbonate has yet to be determined in adult cats. Developing an isotope protocol to achieve equilibrium of ^13^CO_2_ in breath of cats, when L-[1-^13^C]-Phe is used as the tracer, is the next step to allow the successful application of carbon oxidation techniques in this species. Therefore, the aim of the present study was to develop an oral isotope protocol for adult cats that would produce steady state in expired ^13^CO_2_ during the time frame of carbon oxidation studies.

## Materials and methods

The present study was carried out according to the guidelines for animal care and use provided by the Canadian Council on Animal Care. All ethical and animal-related aspects of the pilot trials were approved by the University of Guelph Animal Care Committee (AUP#4424).

### Animal and housing

One adult (2 years old) neutered male purpose bred cat (Marshall Biosciences, North Rose, NY, USA) was used. The cat was housed with other purpose bred cats (*n* 18) in an indoor free-living environment (7⋅1 m × 5⋅8 m) located in the Animal Biosciences Department at the University of Guelph. The room was approved for cat inhabitation by the Chief Veterinary Inspector of the Ontario Ministry of Agriculture, Food and Rural Affairs (OMAFRA) under the Animals for Research Act prior to the arrival of the cats. The environment was enriched with perches, toys, hide boxes, beds, scratching pots and climbing apparatuses. The light (12 h light:12 h cycle), temperature (20 °C) and humidity (40–60 %) were controlled and monitored daily. Cleaning of the litter boxes and exterior surfaces was performed once daily at the same time. Cats were socialised with a familiar individual five days a week, for 2 h each day.

### Study design and diet

The cat used in the study transitioned from a commercial dry diet (T22 Total Grain-Free, Nutram Pet Products, Elmira, ON) to a commercial wet diet (Friskies Paté Salmon Dinner, Purina Wet Cat Food, Purina, St. Louis, MO; Metabolisable energy=1151 kcal/kg; moisture, max = 78 %; crude protein, min = 10 %; crude fat, min = 5 %; crude fibre, max = 1 %, ash, max = 3⋅3 %) over a 6-d period, where the intake of the wet commercial diet gradually increased. The phenylalanine (Phe) and the tyrosine (Tyr) content of the commercial diet were determined via hydrolysis (AOAC, 2012; method 994⋅12) using ultraperformance liquid chromatography (Waters Corporation, Milfor, MA, USA). The cat was then fed 100 % of its daily energy intake to maintain body weight (BW; 269 kcal/d), based on historical feeding and BW records. Food was provided in two equal daily feedings (07:30 and 16:00 h) throughout the study. Water was provided *ad libitum* throughout the study from standing and free-flowing water.

Two separate pilot trials (1 and 2) were conducted. Three isotope protocols (treatments) were tested within each pilot trial. Each treatment was replicated three times using the same cat, totalling three periods. In each period, the order of treatments was randomly assigned. The different isotope and sample collection protocols evaluated in each pilot trial are described below.

#### Pilot trial 1

The objective of this first pilot trial was to determine whether modifying our original isotope protocol^(20)^, by either adding a priming dose of NaH^13^CO_3_ or increasing the priming or constant dose of L-[1-^13^C]-Phe, would result in a steady-state condition. The cat underwent a 2-d feeding regimen: (1) d 0: regular feeding regimen as described above and (2) d 1: IAAO study day where treatments were tested. The cat was fed the same diet through the study as only the effect of isotope protocol on the enrichment of ^13^CO_2_ in breath was being investigated. Thus, the usual 2-d dietary adaption period used in IAAO studies^(23)^ was not required. BW was measured the morning of each IAAO study day to ensure accurate delivery of the isotope dose. This 2-d feeding regimen was repeated 9 times (3) times within each experimental period to achieve three replicates per treatment. A similar IAAO feeding and breath collection protocol as described in our previous study was applied^(20)^. Briefly, three fasting respiration/indirect calorimetry measurements were collected, followed by the feeding protocol. Thirteen meals were offered, corresponding to 50 % of the cat's food allowance; after completing each IAAO, the cat was fed the remaining 50 % of its daily food intake. The first three meals were fed every 10 min (0, 10 and 20 min) to achieve fed state and the following ones were fed every 25 min. Background enrichment was determined by the collection of CO_2_ samples over three consecutive 25 min period after fed state was achieved (45, 75 and 90 min) and before the tracer protocol began. A priming dose of bicarbonate was top-dressed on the sixth meal combined with the priming dose of L-[1-^13^C]-Phe (99 %, Cambridge Isotope Laboratories, Inc., Tewksbury, MA). A constant dose was given simultaneously and continued throughout the remaining meals. Three isotope protocols (treatments) were tested (A, B and C; Table 1). All treatments contained a similar priming dose of NaH^13^CO_3_ (0⋅176 mg/kg) (99 %, Cambridge Isotope Laboratories, Inc., Tewksbury, MA). The priming dose of NaH^13^CO_3_ was derived based on the priming dose utilised in IAAO studies in humans^(22)^. Treatment A followed the priming (4⋅8 mg/kg) and constant (1⋅04 mg/kg) doses of L-[1-^13^C]-Phe as we previously used^(20)^ to determine whether the failure to achieve ^13^C steady state in the breath of cats^(20)^ was due to an improper prime to constant ratio of L-[1-^13^C]-Phe or simply due to the need to prime the CO_2_ pool prior to L-[1-^13^C]-Phe provision. In treatments B and C, the priming (9⋅4 mg/kg) and constant (2⋅4 mg/kg) dose of L-[1-^13^C]-Phe were increased, respectively, based on the doses used for dogs^(15,24)^.

**Table 1.** Isotope protocol for pilot trials 1 and 2

Isotope* (mg/kg)	Pilot trial 1^†^				Pilot trial 2^‡^			
	Meal	A	B	C	Meal	D	E	F
Prime NaH^13^CO_3_	6th	0⋅176	0⋅176	0⋅176	4th	0⋅264	0. 352	0⋅44
Prime L-[1-^13^C]-Phe	6th	4⋅8	9⋅4	9⋅4	5th	4⋅8	4⋅8	4⋅8
Constant L-[1-^13^C]-Phe	6–13th	1⋅04	1⋅04	2⋅4	5–13th	1⋅04	1⋅04	1⋅04

NaH^13^CO_3_: ^13^C-Sodium bicarbonate; L-[1-^13^C]-Phe: L-[1-^13^C]-Phenylalanine.

* Isotope dosing solutions were top-dressed on the respective meals.

† Fed background of ^13^CO_2_ (*n* 3) was determined by collecting breath samples after the third, fourth and fifth meals.

**Fig. 1.**
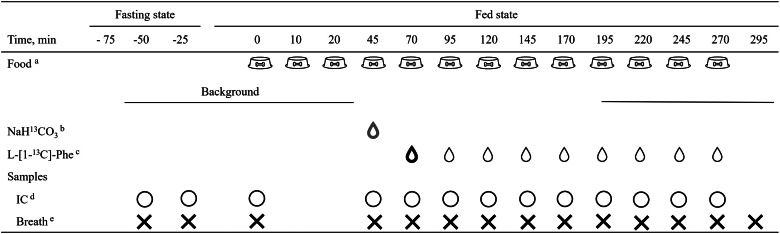
Feeding, isotope and sample protocol (treatment F in pilot trial 2) proposed to be utilised in IAAO studies in cats. ^a^Each meal represented one-thirteenth of half of the daily food intake for the cat. ^b^Priming dose of NaH^13^CO_3_ was top-dressed on the fourth meal (time: 45 min). ^c^Priming dose of L-[1-^13^C]-Phenylalanine (L-[1-^13^C]-Phe) was top-dressed on the fifth meal. The continuous dose of L-[1-^13^C]-Phe started on the fifth meal with the priming dose, followed by continuous supply through the remaining meals. ^d^IC: indirect calorimetry. Three 25-min measures of respiratory gases were obtained prior to feeding to obtain the resting volume of CO_2_ produced (VCO_2_). Starting at 45 min, VCO_2_ was measured in 25-min intervals for the duration of the study. ^e^Three 25-min breath samples collection for ^13^CO_2_ background were obtained at −50, −25 and 0 min (fasted state) before food and isotope provision. One breath sample was collected at time −45 min before isotope provision for determination of ^13^CO_2_ background during fed state. Breath samples were then collected every 25 min for the duration of the study.

‡ Fasted background of ^13^CO_2_ (*n* 3) was determined by collecting breath samples before meal provision (time: −50, −25 and 0 min) and fed background of ^13^CO_2_ (*n* 1) was determined by collecting one breath sample after the third meal (time: 45 min). The detailed protocol is presented in Fig. 1.

#### Pilot trial 2

Having established the ideal prime and constant doses of L-[1-^13^C]-Phe, we proceed to pilot 2, in which we aimed to determine the ideal priming dose of NaH^13^CO_3_. The same 2-d feeding regimen was used as described above. During IAAO, the feed regimen was kept similar, but the time of isotope provision and breath sample collection for determination of background of ^13^CO_2_ was modified (Fig. 1). Three breath samples were collected prior to feeding to determine the fasted background of ^13^CO_2_ (−50, −25 and 0 min), and one breath sample was collected after fed state was achieved (45 min) to determine the fed background of ^13^CO_2_. Three isotope protocols were tested (D, E and F; Table 1). Three priming doses of NaH^13^CO_3_, top-dressed on the fourth meal, were tested (D: 0⋅264 mg/kg; E: 0⋅352 mg/kg; F: 0⋅44 mg/kg), while the priming (4⋅8 mg/kg) and constant (1⋅04 mg/kg) doses of L-[1-^13^C]-Phe were kept similar across treatments. The priming dose of L-[1-^13^C]-Phe was top-dressed on the fifth meal and a constant dose was given simultaneously and continued throughout the remaining meals.

### Breath samples analysis

Samples of CO_2_ were collected by trapping subsamples of expired CO_2_ in 8 ml of 1M NaOH over 25-min periods. The samples were transferred and retained in a 10 ml vacutainer tube (#366430 BD) that was evacuated to prevent dilution of ^13^CO_2_ and stored at room temperature until analysis. Analysis of ^13^C enrichment in breath CO_2_ samples was done at the Environmental Isotope Laboratory, University of Waterloo (200 University Ave W, Waterloo, ON, Canada) using a Gasbench II interfaced with a Delta V Plus mass spectrometer (Thermo Scientific, Bremen, Germany). Enrichments were expressed above background samples (Atom percent excess, APE).

### Statistical analysis

A sample size of one is commonly utilised to assess the dynamics of metabolites *in vivo* in pilot trials^(27)^. Thus, a single cat was utilised in pilot trials 1 and 2 to comply with the three Rs principle of animal experimentation^(28)^. Treatments were replicated using the same cat to account for variation between days. Isotopic steady state was defined as the enrichment of ^13^CO_2_, as APE, remaining constant in at least the last three breath samples. The APE was fitted against meal number (offered in 25-min intervals) to determine the number of meals necessary, within each isotope protocol, to achieve steady state of ^13^CO_2_. Steady state was evaluated by visual inspection, by regression analysis using add-in Analysis ToolPak in Microsoft Office Excel 2020 and by competing statistical models, namely broken-line linear (BLL) or broken-line quadratic (BLQ) model using PROC NLMIXED in SAS (SAS Inst., Cary, NC). Models were compared based on the Bayesian information criterion (BIC), where the smaller the value, the better the fit to the model^(29)^. Differences between fasted and fed background enrichments in pilot trial were analysed using PROC GLIMMIX with physiological state (fasted *v.* fed) as the fixed effect. Statistical difference was declared when *P* < 0⋅05.

## Results

**Fig. 2.**
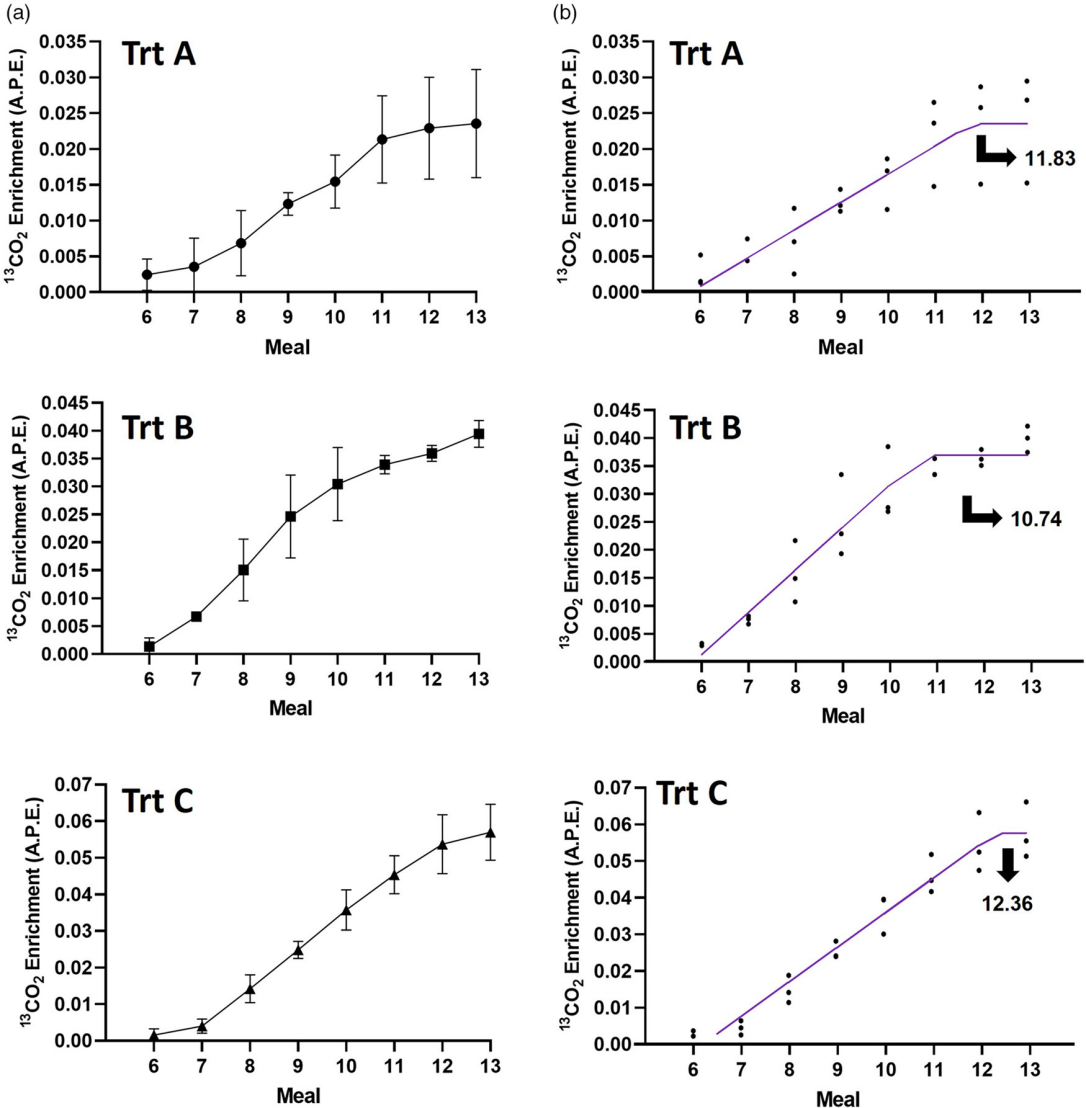
Pilot trial 1: (a) visual inspection (values are *x̄* ± sd) and (b) fitted broken-line linear model for ^13^CO_2_ expressed as atoms percent excess (APE) as a function of meal (25-min intervals). Isotope was provided orally over small meals. The priming dose (0⋅176 mg/kg) of NaH^13^CO_3_ remained similar among treatments (Trt), while the priming and constant doses of L-[1-^13^C]-Phe varied as follows. Trt A: priming dose: 4⋅8 mg/kg; constant dose: 1⋅04 mg/kg. Trt B: priming dose: 9⋅4 mg/kg; constant dose: 1⋅04 mg/kg. Trt C: priming dose: 9⋅4 mg/kg; constant dose: 2⋅4 mg/kg.

**Fig. 3.**
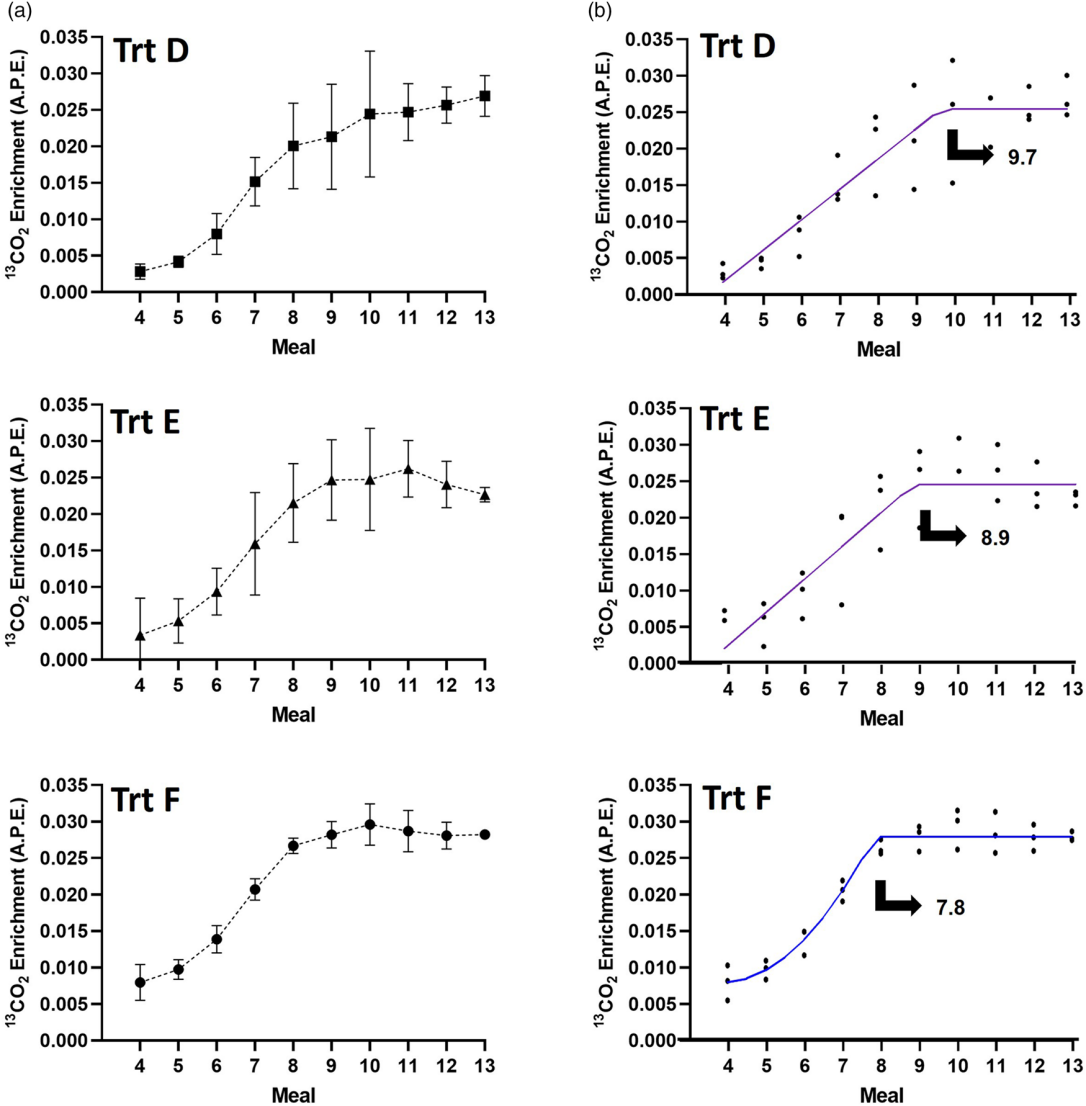
Pilot trial 1: (a) visual inspection (values are *x̄* ± sd) and (b) fitted broken-line linear (purple) or broken-line quadratic (blue) model for ^13^CO_2_ expressed as atoms percent excess (APE) as a function of meal (25-min intervals). Isotope was provided orally over small meals. The priming (4⋅8 mg/kg) and constant (1⋅04 mg/kg) doses of L-[1-^13^C]-Phe remained similar among treatments (Trt). The priming dose of NaH^13^CO_3_ varied across Trt D (0⋅264 mg/kg), Trt E (0⋅352 mg/kg) and Trt F (0⋅44 mg/kg).

The cat remained healthy and maintained BW throughout both pilot trials (data not shown). In every IAAO study day, all meals were consumed immediately after each feeding. In pilot trial 1, the slope of the line for breath ^13^CO_2_ enrichment data for the last three samples was not significantly different from zero for treatment A (*P* = 0⋅14), B (*P* = 0⋅10) and C (*P* = 0⋅16). The coefficient of variation (CV) for the last three samples was the lowest for treatment A (5⋅06 %) followed by treatments B (7⋅65 %) and C (11⋅59 %), which are considered high CV for plateau enrichment of ^13^CO_2_^(8)^. Thus, even though the slope was not significantly different from zero, we did not feel confident to declare that steady state was achieved due to the high CV and as enrichment was still rising through numerical and visual inspection (Fig. 2). Thus, BLL and BLQ analysis were performed to provide an additional method to quantitatively assess isotopic plateau in CO_2_. The model that best fit the enrichment of ^13^CO_2_ was the BLL for all treatments in pilot 1 (lowest BIC). The breakpoints estimated occurred at approximately meal 12 for treatments A and C and at meal 11 for treatment B (Fig. 2). In pilot 2, the slope of the line for breath ^13^CO_2_ enrichment data for the last three samples was significantly different from zero (*P* = 0⋅04) in treatment D, but it was not in treatments E (*P* = 0⋅08) and F (*P* = 0⋅49). The CV for the last three samples for treatment F was the lowest (1⋅11 %) followed by D (4⋅32 %) and E (7⋅43 %). The model that best fit the enrichment of ^13^CO_2_ was the BLL for treatments D and E and BLQ for treatment F. The asymptote occurred at approximately meals 10, 9 and 8 for treatments D, E and F, respectively (Fig. 3). No differences (*P* = 0⋅30) were observed in fasted and fed background enrichment of ^13^CO_2_ (1⋅102 *v.* 1⋅101 % ± 0⋅001, least square means ± sem) evaluated in pilot trial 2.

## Discussion

The present study was conducted to develop an oral isotope infusion protocol in cats that would produce steady-state conditions of expired ^13^CO_2_ for subsequent carbon oxidation studies, such as IAAO. In the IAAO methodology, phenylalanine (Phe) meets the criteria to be used as the indicator AA^(30)^, and thus, L-[1-^13^C]-Phe is the tracer of choice to measure flux of ^13^CO_2_ at varying intakes of test AA. When Phe is used as the indicator AA, Tyr must be provided in excess to ensure that changes in Phe oxidation are solely due to changes in the intake of the test AA and are not being used to obtain the metabolic requirement for Tyr^(31)^. The diet contained 0⋅82 and 1⋅46 % of Phe and Phe + Try on a dry matter basis, respectively, supplying almost twice the requirement established by the Association of American Feed Control Officials^(32)^ for adult cats consuming commercial diets (Phe = 0⋅45 % and Phe + Try = 0⋅74 %; dry matter basis). Furthermore, dietary Phe (including the intake of the tracer) also needs to be similar among dietary treatments^(30)^ or pool size will differ. Unlike our previous isotope dilution study^(20)^, where (in each experimental day) cats were fed thirteen small meals corresponding to their total feed allowance, only half of the daily feed allowance was provided in each IAAO day in the present study to ensure that all small meals were promptly consumed. This feeding regimen is commonly applied in IAAO studies in pigs^(23,33–35)^ and does not affect the metabolic outcome of interest because the ratio of indispensable AA consumed is not affected by the meal size. While the flux of Phe is affected by its dietary intake, the % of L-[1-^13^C]-Phe that is oxidised should be similar whether half or the total daily feed allowance is provided during IAAO studies, not affecting the breakpoint. Thus, the results observed herein can be solely attributed to perturbations in the kinetics of Phe and/or bicarbonate owing to different isotope dosages.

In pilot trial 1, increasing the priming dose (9⋅4 mg/kg) of L-[1-^13^C]-Phe or increasing the priming (9⋅4 mg/kg) together with the constant dose (2⋅4 mg/kg) did not result in steady state of ^13^CO_2_ in breath samples. Although the latter isotope protocol was successful in producing a steady-state condition of ^13^CO_2_ in breath of dogs during IAAO studies^(15,24,36–39)^, the enrichment of ^13^CO_2_ indicates that the Phe pool was overprimed in the present study. Over-priming results in a negative slope following the initial rise in enrichment. If breath samples had been collected for longer periods, the negative slope would likely be detected, and thus, a longer period would be required to achieve steady state. Thus, we hypothesised that the priming and constant doses of 4⋅8 and 1⋅04 mg/kg of L-[1-^13^C]-Phe, respectively, are ideal for cats. Likely, cats failed to achieve steady state of ^13^CO_2_ in breath when this isotope protocol was used in our previous study^(20)^ due to a lack of priming of the bicarbonate pool. In parentally fed human neonates, the isotopic steady state of ^13^CO_2_ in breath was achieved 12 h after the start of L-[1-^13^C]-Phe infusion without provision of a priming dose of NaH^13^CO_3_^(40–42)^. Likely, steady state of ^13^CO_2_ in breath of cats would have been achieved with a longer feeding regimen and continuous supply of L-[1-^13^C]-Phe. It would be difficult, however, to apply such a 12 h half-hourly feeding regimen in cats as they are not parentally fed, and we rely on their continuous voluntary food intake to successfully apply the IAAO protocol. Although priming the bicarbonate pool in pilot trial 1 (treatment A) improved the response in ^13^CO_2_, the dose applied (0⋅176 mg/kg) [which is the same previously used in children^(43)^, men^(22)^ and women^(44)^] was not ideal for cats as changes in enrichment of ^13^CO_2_ at the last data points were still observed, and thus, higher priming doses of NaH^13^CO_3_ were tested in the following pilot trial (pilot trial 2). We also administered the NaH^13^CO_3_ dose prior to the priming dose of L-[1-^13^C]-Phe to enrich the bicarbonate pool in advance, allowing for ^13^CO_2_ from oxidation of Phe to be detected. Steady-state ^13^CO_2_ enrichment in the cat's breath was maintained and achieved faster when the highest priming dose of NaH^13^CO_3_ (0⋅44 mg/kg) was provided compared to the other doses (0⋅176, 0⋅264 and 0⋅352 mg/kg). The priming dose considered ideal for cats (0⋅44 mg/kg) is 2⋅5 times higher than the dose used in IAAO studies in humans^(22,43,44)^, indicating a greater retention of CO_2_ and slower appearance of CO_2_ into, and transit through, the bicarbonate pool. This finding was unexpected because cats, due to their smaller size than humans, would have a higher flux between labelled ^13^CO_2_ and the unlabelled CO_2_ in the bicarbonate pool^(21)^, which should have necessitated smaller doses of labelled NaH^13^CO_3_ to achieve steady state. As such, the high NaH^13^CO_3_ priming dose we observed in the cat indicates a larger bicarbonate pool or greater retention of CO_2_, separate from the bicarbonate pool, arising from idiosyncrasies inherent to this species and this finding deserves further research.

**Fig. 4.**
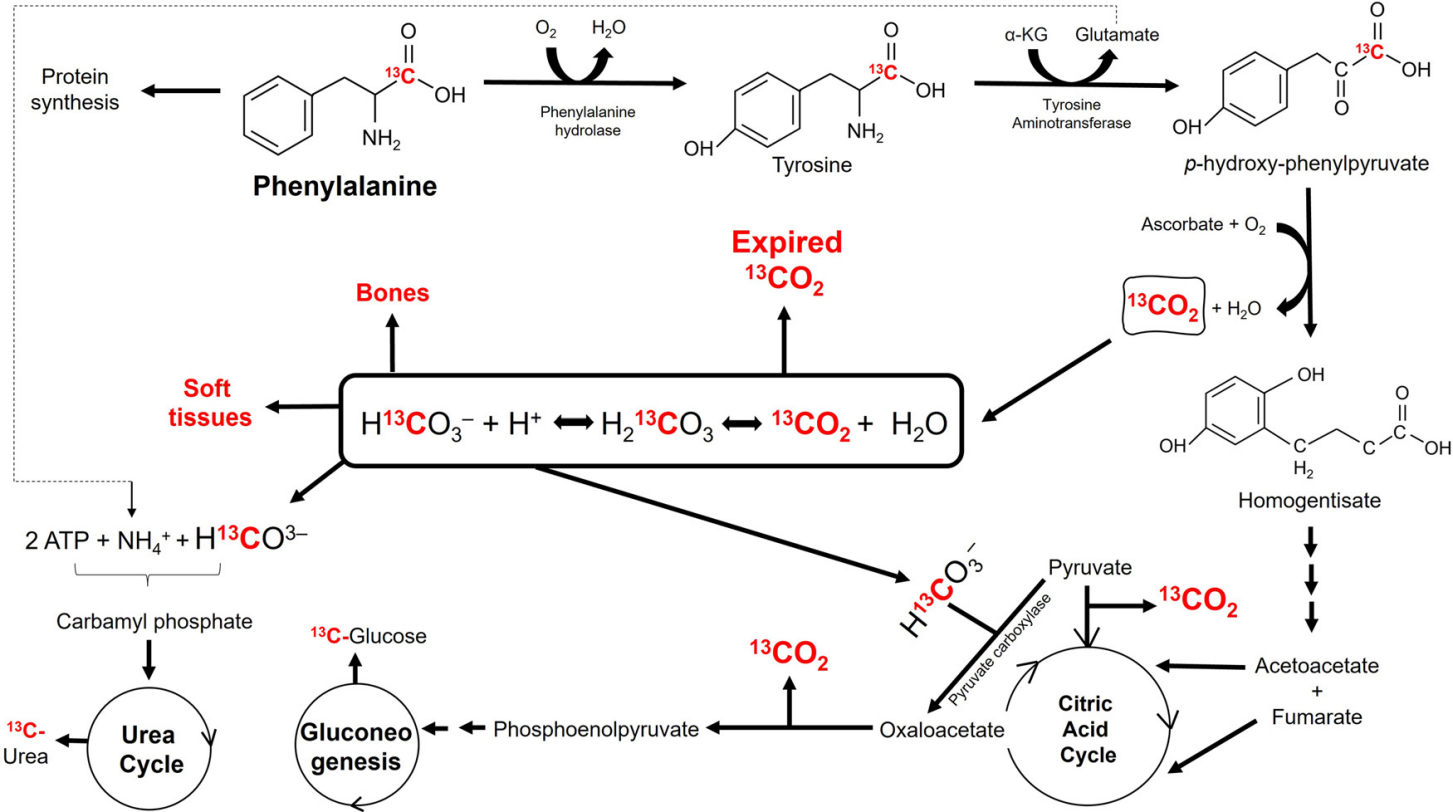
Major metabolic fates of ^13^CO_2_ derived from oxidation of L-[1-^13^C]-phenylalanine.

Cats are obligatory carnivores, and their metabolism has adapted to a diet consisting predominantly of animal tissues, with many of the adaptations relating to the carbohydrate and protein content of the diet^(45)^. Cats have a higher requirement for nitrogen (N) due to their limited ability to regulate AA catabolic enzymes^(46)^. Furthermore, cats do not reduce the activity of urea cycle enzymes^(46)^ when fed lower protein diets like omnivorous and herbivorous animals^(47–50)^. The first step of the urea cycle is to convert ammonia (product of AA oxidation) and bicarbonate to carbamoyl phosphate. When NaH^14^CO_3_ was intravenously injected in a cat, 98 % of the urea carbon synthesised was derived from labelled bicarbonate, with 50 % of the bicarbonate incorporation into urea occurring in the first 30 min^(51)^. This indicates that the high priming dose of NaH^14^CO_3_ necessary to achieve a steady state of ^13^CO_2_ in the breath of cats may be due to greater utilisation of bicarbonate by the urea cycle (Fig. 4). Measuring enrichment of ^13^C in urea would indicate losses of ^13^CO_2_ via the urea cycle. Unfortunately, enrichment of ^13^C was only measured in breath samples in the present study due to difficulty in sample collection. Furthermore, at least half of the incorporation of ^14^C in the urea cycle occurred rapidly, 30 min post-prime, with NaH^14^CO_3_ provision^(51)^. Priming the bicarbonate pool prior to priming the pool of the substrate of interest (e.g., Phe) might, to some extent, allow earlier detection of ^13^CO_2_ derived from the oxidation of L-[1-^13^C]-Phe, and thus, help to achieve ^13^CO_2_ steady state in breath faster.

The lack of ability to control AA catabolic enzymes may be beneficial for cats as AA are used for gluconeogenesis. The activity of the rate-limiting enzyme of gluconeogenesis (e.g., pyruvate carboxylase) in the liver is upregulated in cats compared to dogs^(52)^. The conversion of pyruvate to oxaloacetate via pyruvate carboxylase also requires bicarbonate as a substrate, and thus, would be a reason for retention of CO_2_ to be used in the process of gluconeogenesis (Fig. 4). Indeed, the metabolic status of rapid gluconeogenesis may affect the recovery of labelled CO_2_. A lower recovery of ^14^CO_2_ in NaH^14^CO_3_-perfused livers of starved compared to fed rats^(53)^ was observed and is likely due to a higher rate of gluconeogenesis in the former. The dietary macronutrient content may also affect recovery of labelled CO_2_, with a greater intake of carbohydrates resulting in reduced gluconeogenesis, and thus, affecting the NaH^13^CO_3_ dose. The same authors^(53)^ also observed a net incorporation of ^14^CO_2_ into other compounds, such as urea, protein, and carboxylic and amino acids. The rate of incorporation into each substrate varies widely and it is affected by metabolic state^(54)^; thus, animals need to be in the same metabolic state and to maintain their BW throughout IAAO studies. A fraction of CO_2_ that is not recovered in breath may also be lost in slowly exchanging pools, such as bone (Fig. 4). In the cat, approximately 6 % of injected NaH^14^CO_3_ was found in the bone after 4 h, while only 1 % was found in muscles, viscera and blood^(51)^. It is also important to consider that the bicarbonate pool is not homogeneous and the rate of excretion of labelled carbon after intravenous injection of labelled bicarbonate follows an exponential equation with three major bicarbonate pools with distinct kinetics: a central vascular pool, a rapid turnover pool (bicarbonate in soft tissues) and a slow turnover pool (bicarbonate in bone tissue)^(54–56)^. Furthermore, CO_2_ transport and storage dynamics are sensitive to changes in acid-base status^(57)^; thus, dietary electrolyte balance should be considered in future carbon oxidation studies. While it has been recently reported that changes blood pH via dietary supplementation can affect protein kinetics in humans^(58)^, this has yet to be investigated in cats. As the intake of some AA can influence acid-base balance^(59)^ and in dose-response studies concentrations of the test AA are provided in graded levels, caution should be taken to ensure a consistent electrolyte composition among test diets.

To account for retention of labelled CO_2_, the bicarbonate retention factor (BRF) is usually determined and used as a correction factor to compute the flux of CO_2_ in carbon oxidation studies. Although determining the BRF in cats would aid our understanding pertaining the metabolism of bicarbonate in this species, the goal of the present study was to determine the best isotopic protocol to achieve steady state of ^13^CO_2_ in breath samples of cats when L-[1-^13^C]-Phe is used as the tracer in IAAO studies. The BRF used to compute flux of ^13^CO_2_ does not influence the achievement of steady state of ^13^CO_2_ in breath, only the accuracy of the total ^13^CO_2_ excretion. We used the BRF determined in dogs which assumes 100 % recovery of ^13^CO_2_, and thus, likely underestimates the total absolute flux of ^13^CO_2_ in cats. The BRF, however, does not influence the degree of change of flux of ^13^CO_2_, which is more important than the absolute value in oxidation studies. As mentioned above, CO_2_ retention can be influenced by factors inherent to the animal (e.g., metabolic status) and the diet (e.g., electrolytic composition); thus, ideally, one should determine the BRF under conditions identical to the isotope study and using the same system for breath collection and measurement of respiratory exchange^(60)^ as done previously in dogs^(15)^ and chickens^(13)^. Given the numerous factors influencing BRF, future studies should investigate the effects of properties of the diet (e.g., electrolytic composition and protein content) on the acid balance of the cat and, consequently, on bicarbonate retention.

## Conclusions

An isotopic steady state of ^13^CO_2_ enrichment in breath can be achieved in cats using a thirteen small meal regimen; wherein a priming dose of NaH^13^CO_3_ (0⋅44 mg/kg) and L-[1-^13^C]-Phe (4⋅8 mg/kg) should be provided in the fourth and fifth meals, followed by a constant dose (1⋅04 mg/kg) of L-[1-^13^C]-Phe in the next meals. Fasted background of ^13^CO_2_ can be used if there are no major differences in the macronutrient composition of dietary treatments. This protocol resulted in an isotopic steady-state condition necessary to successfully use the IAAO technique in cats, which can be used to determine indispensable AA requirements and AA bioavailability in future studies.
